# Factors associated with uptake of *Haemophilus influenzae* type b vaccination in Shanghai, China

**DOI:** 10.1186/s12887-018-1374-6

**Published:** 2019-01-07

**Authors:** Ya Yang, Yingjian Wang, Dongjian Yang, Shurong Dong, Yu Yang, Xu Zhu, Yue Chen, Yibiao Zhou, Qingwu Jiang

**Affiliations:** 1 Key Laboratory of Public Health Safety, Ministry of Education, Tropical Disease Research Center, Department of Epidemiology, School of Public Health, Fudan University, 130 Dong’an Road, Shanghai, 200032 China; 2 China Office of United Nations Children’s Fund, Beijing, China; 3 School of Epidemiology and Public Health, Faculty of Medicine, University of Ottawa, Ottawa, Canada

**Keywords:** *Haemophilus influenzae type b* vaccine, Coverage, Timeliness, Completion

## Abstract

**Background:**

*Haemophilus influenzae type b* (Hib) vaccine is effective in reducing the burden of Hib related diseases, but little is known about factors influencing the uptake of Hib vaccine. This study aimed to assess the uptake of Hib vaccination and its associated factors in Shanghai City, China.

**Methods:**

We used data from a retrospective cohort of 183,246 children born in 2012–2016 obtained from the Shanghai Immunization Program Information System, which provided information on the uptake of Hib vaccination. We conducted a cross-sectional study of 451 children to collect information on demographic and other factors that might be associated with Hib vaccination.

**Results:**

In the retrospective cohort study, the proportions of Hib dose-1 coverage, vaccination completion and timeliness were 67.7, 52.2 and 29.4%, respectively. These measures were better among local children and increased with birth year, while there were regional differences. Hib vaccine uptake was significantly associated with maternal occupation (non-health vs health workers, OR = 2.33, 95% CI: 1.32–4.13, *P* = 0.004) and caregivers’ awareness of Hib (yes vs no, OR = 1.75, 95% CI: 1.12–2.74, *P* = 0.013).

**Conclusions:**

We found low levels of coverage of dose-1 Hib vaccine, timeliness and completion, suggesting inadequate protection against Hib disease for children in Shanghai. Non-local children and those of health workers should be targeted for interventions. The inclusion of Hib vaccine into the national immunization program could help improve the uptake of Hib vaccines.

## Background

*Haemophilus influenzae type b* (Hib) is an important bacterial cause of meningitis, pneumonia and other severe infections including bacteremia, cellulitis, epiglottitis, septic arthritis, osteomyelitis and pericarditis in children worldwide [1, 2]. In 2000, at which point Hib vaccine was not routinely offered in many low- or middle-income countries, Hib caused about 8.13 million cases of serious disease and 371,000 deaths in children aged 1–59 months globally [2]. As most countries were using Hib vaccine at the end of 2015, 29,500 HIV-uninfected children aged 1–59 months died of Hib diseases that year [3]. For Hib meningitis, 9.5% of patients with adequate medical treatment ended up with at least one major severe sequela such as blindness, epilepsy, deafness, and/or learning disabilities [4].

Highly effective and safe Hib conjugate vaccines can prevent diseases caused by Hib. The World Health Organization (WHO) recommends that Hib vaccination be included in all routine infant immunization programs in 2013 [1]. The inclusion of Hib conjugate vaccine in routine infant immunization programmes has led to significant and sustained reductions in childhood Hib morbidity and mortality in Kenya [5], France [6], and Denmark [7]. Routine use of Hib vaccine also reduced the nasopharyngeal carriage prevalence of Hib and the risk of transmission of Hib between individuals in Kenya [5] and Bangladesh [8].

The Hib conjugate vaccine was licensed in China in 1997. A recent meta-analysis found that a declining trend of respiratory tract infections caused by Hib was associated with the introduction of the Hib vaccine; however, the carriage of Hib among children changed little [9]. Five monovalent and four combined Hib vaccines had been issued during the period of 2015–2017 based on data from China’s National Institute for Food and Drug Control. In China, vaccines are classified into Category I and II vaccines. Category I vaccines are funded by the government and offered to all children free of charge while the Category II vaccines are administered voluntarily and must be paid for by parents or caregivers. Hib vaccine was ranked the second most widely used Category II vaccine with an estimated coverage rate of 46.7% in P.R. China in 2014 [10]. Shanghai is the largest and most developed city in China and holds a population of more than 24 million, including 9.7 million non-local residents, according to the Shanghai Statistical Yearbook 2017 [11]. The non-local children merited special attention because of low coverage for self-paid vaccines [12, 13]. A low rate of Hib vaccination accompanied by an extremely low level of timely dose 1 vaccination was observed in children aged 2–7 years in Shanghai in 2012 [13] Factors influencing the uptake of Hib vaccine has not been well understood. The aim of our study was to provide an update on the Hib vaccine coverage in Shanghai and to determine influencing factors.

## Methods

### Design

The current study included 2 urban districts (Changning and Jing’an) and 1 suburban one (Jiading) of Shanghai. Jing’an is one of the central and most densely populated districts, with an area of 37.4 km^2^ and a population of 1.07 million in 2017. Changning, which borders Jing’an to the east and Jiading to the west, has a land area of 38.3 km^2^ and a population of 0.69 million by the end of 2017. Jiading is a suburb, with a population of 1.58 million in 2017 and an area of 463.6 km^2^. These three districts were good representatives for Shanghai city based on health authorities’ opinions and had full functioning immunization information systems. In a retrospective cohort study, we used the Shanghai Immunization Program Information System (SIPIS) to obtain Hib conjugate vaccine utilization data for the three districts.

We also conduct a cross-sectional study in the three districts to assess Hib vaccine uptake and its determinants. Our survey targeted parents or caregivers who accompanied their child for a vaccination visit and the child was between 2 months and 7 years of age. Assuming a 50% acceptance of Hib vaccination for children and 5% confidence limits, we calculated a sample size of 385 study participants. Considering a small number of invalid questionnaires, we opted for 450 participants, with 150 from each district. Participants were consecutively recruited at the immunization clinics.

### Data collection

#### Hib vaccine coverage

The SIPIS has all records for the administration of both NIP and non-NIP (for-fee) vaccines in the three selected districts. A cohort of children born from January 1, 2012 through December 31, 2016 from the SIPIS had the following information: sex, birth date, district of residence, residency status (local or non-local population), and receipt of Hib vaccine (date of receipt and type of Hib vaccine).

Since Hib vaccines were from different manufacturers with varied recommended vaccination schedules, three vaccination outcomes were assessed in the study: dose-1 vaccination, completion, and timeliness of dose-1 vaccination. Dose-1 vaccination was characterized as the proportion of children who had received an initial dose of Hib vaccine. Vaccine completion was calculated as the proportion of children with dose-1 vaccination who completed the series recommended by manufactures. For children with an initiation of Hib vaccination at < 1 year, a series completion required 2 primary doses as well as a booster after 1 year of age. For those who had the first dose administered at ≥1 year of age, only 1 additional dose was needed for completion. Timeliness of dose-1 vaccination was characterized as the proportion of children who received their first Hib vaccine at the age of 38 to 123 days.

#### Questionnaire survey

The cross-sectional survey was conducted in April 2017. Health staff invited eligible parents or caregivers to participate in the survey during their visit at community immunization clinics of the three districts of Shanghai. Participants were informed of the purpose of the study and completing the questionnaire was voluntary. All participants gave informed consent prior to the survey. The questionnaire consisted of participants’ demographics, awareness of Hib and Hib vaccination, and immunization status of their children.

### Statistical analysis

Data were exported from the SIPIS and carefully cleaned, with incomplete cases removed. Vaccination status was compared using Chi-square test. In case of zero counts, Fisher’s exact test was used for significance test. Cochran-Armitage test was used to identify the temporal trend of Hib vaccination. Reciprocal Kaplan-Meier curves were generated for the cumulative incidence of Hib dose-1 vaccination.

We used Pearson’s chi-squared test to assess the associations between Hib vaccine uptake and its determinants. Fisher’ s exact test was used when appropriate. Variables with a *P* value < 0.10 in the bivariate analysis were included in multivariable analysis. Final models included variables that remained statistically significant (*P*-value ≤0.05). Odds Ratios (ORs) and their 95% confidence intervals (95% CIs) were calculated. All analyses and drawing were performed using R software version 3.3.1.

## Results

### Coverage, timeliness and completeness of Hib vaccination – A retrospective cohort study

We identified a total of 183,246 records from the SIPIS (Table 1). Most individuals were from Jiading district (69.4%), 65.1% were not permanent residents (non-local population) and 52.8% were boys.

**Table 1:** Characteristics and Hib vaccination outcomes for children born during 2012–2016

	No. (%)	Dose-1 Hib vaccination, %^a^	Completion, %^a^	Timeliness of dose-1, %
Total	183,246	67.7	52.5	29.4
District				
Jiading	127,104 (69.4)	67.5	56.2	29.4
Jing’an	29,157 (15.1)	68.4	42.0	30.0
Changning	26,985 (14.7)	67.5	46.5	29.1
*P value*		< 0.001	< 0.001	0.048
Birth year				
2012	35,998 (19.6)	63.9	49.1	23.7
2013	36,593 (20.0)	68.5	51.2	25.7
2014	39,832 (21.7)	69.3	55.1	27.3
2015	32,731 (17.9)	68.9	54.5	32.1
2016	38,092 (20.8)	–	–	38.2
*P for trend*		< 0.001	< 0.001	< 0.001
Sex				
Male	96,665 (52.8)	67.8	52.5	29.1
Female	86,581 (47.2)	67.5	52.5	29.7
*P value*		0.268	0.966	0.003
Residency				
Local	63,932 (34.9)	68.4	55.0	45.3
Non-local	119,314 (65.1)	67.3	51.2	20.9
*P value*		< 0.001	< 0.001	< 0.001

^a^Calculated for children born during 2012–2015

**Fig. 1:**
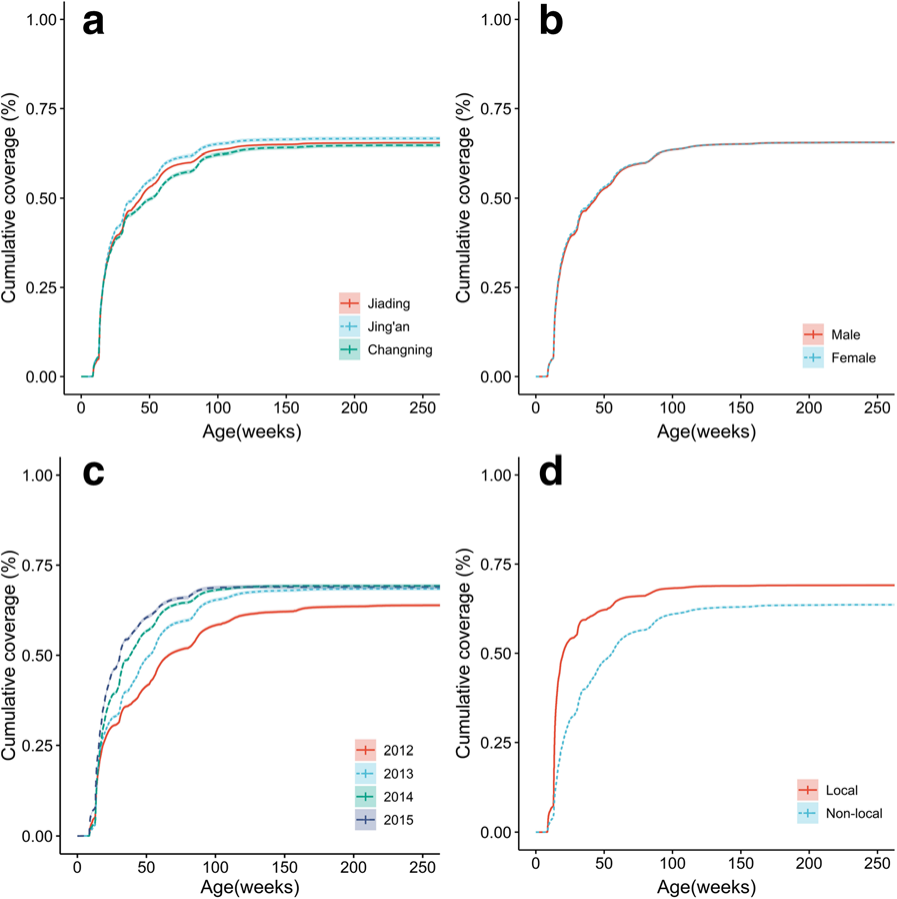
Cumulative coverage of Hib vaccine dose-1 by district (**a**), sex (**b**), birth year (**c**) and district of residence (**d**)

Table 1 also shows the coverage, timeliness and completeness of Hib vaccination. Overall, the proportion of Hib dose-1 vaccination was 67.7%. Of children who received at least one Hib dose, the mean age at vaccination was 32.0 weeks (median: 19.3; IQR: 13.9–41.7). 52.9% of children were vaccinated by 50 weeks of age. There was a significant difference among the study districts (*P* < 0.001; Fig. 1a). The highest dose-1 coverage was achieved in Jing’an (68.4%), followed by Jiading (67.5%) and Changning (67.5%). We observed no sex-difference for the dose-1 coverage (Fig. 1b). Local children with a status of permanent residence (68.4%) had a higher coverage than the non-locals (67.3%) (*P* < 0.001; Fig. 1d). An increasing trend of coverage was observed over the study years (*P* for trend< 0.001; Fig. 1c).

The completion rate of Hib vaccination was 52.5% for children born during 2012–2015 (Jiading: 56.2%; Changning: 46.5%; Jing’an: 42.0%), and was higher in local (55.0%) than non-local dwellers (51.2%) (*P* < 0.001). The completion rate increased with birth year from 2012 to 2015 (*P* for trend< 0.001) (Table 1).

Only 29.4% of children received a timely dose-1 Hib vaccine. The proportion of timely vaccination was 30.0% in Jing’an, 29.4% in Jiading and 29.1% in Changning. Timely administration was more frequent in local (45.3%) than non-locals (20.9%) children (*P* < 0.001). Timeliness of dose-1 Hib vaccination improved with birth year (*P* for trend< 0.001) (Table 1).

The coverage of dose-1 Hib vaccine increased with birth year in all districts, and was higher in local than non-local children in Jiading and Jing’an but not in Changning. Series completion increased with birth year in Jiading, but not in Jing’an and Changning. Local children had a higher completion rate than non-local children in Jiading, but not in Changning and Jing’an. There was a significant and steady increase in timeliness of dose-1 Hib vaccination among children in three districts. Local children tended to have a better rate of timeliness than non-local children.

### Hib vaccine uptake and associated factors – A cross-sectional study

Of 452 respondents, one did not complete the questionnaire. Sociodemographic characteristics of children and their parents are shown in Table 2. About half of their children were female (50.6%). The majority of children were of Han ethnicity (98.2%). Most of them had no allergy (87.6%) and 82.0% were aged younger than 30 months. Approximately 60% of mothers and 70% of fathers were aged 30–39 years, and more than 80% of them received college or higher education. Approximately half had a household annual income of ≥200 thousand Yuan, and 44.3% were aware of Hib.

**Table 2:** Analysis of factors associated with Hib vaccine uptake

Variables	Total participants	No. vaccinated (%)	Bivariate analysis	Multivariate analysis	
			*P* value	aOR (95% CI)	*P* value
Child characteristics					
Overall	451	332(73.6)			
Sex			0.644		
Female	228	170(74.6)			
Male	223	162(72.6)			
Age (months)			0.111^a^		
1–11	204	146(71.6)			
12–23	102	81(79.4)			
24–35	64	50(78.1)			
36–47	43	32(74.4)			
48–59	26	18(69.2)			
≥ 60	12	5(41.7)			
Ethnicity			0.687^a^		
Han	443	325(73.4)			
Others	8	7(87.5)			
Resident district			0.021		
Jiading	136	110(80.9)			
Jing’an	125	85(68)			
Changning	124	95(76.6)			
Others	66	42(63.6)			
Having allergy			0.692		
No	395	292(73.9)			
Yes	56	40(71.4)			
Parent characteristics					
Mother age (years)			0.477^a^		
20–29	170	130(76.5)			
30–39	266	191(71.8)			
40–49	12	8(66.7)			
Mother education			0.907		
Middle or high school	80	60(75.0)			
College	304	222(73.0)			
Post graduate or higher	64	48(75.0)			
Mother occupation			0.010		
Health workers	63	38(60.3)		1.00	
Non-health workers	387	293(75.7)		2.33(1.32–4.13)	0.004
Father age (years)			0.582		
20–29	96	70(72.9)			
30–39	319	238(74.6)			
≥ 40	36	24(66.7)			
Father education			0.259		
Middle or high school	86	64(74.4)			
College	284	214(75.4)			
Post graduate or higher	80	53(66.3)			
Father occupation			0.024^a^		
Health workers	441	328(74.4)			
Non-health workers	10	4(40.0)			
Family annual income (1000 Yuan)			0.278		
≤ 9	60	46(76.7)			
10–19	173	133(76.9)			
≥ 20	218	153(70.2)			
Being aware of Hib			0.036		
No	251	175(69.7)		1.00	
Yes	200	157(78.5)		1.75(1.12–2.74)	0.013
Caregivers being informed of Hib vaccines			< 0.001^a^		
No	31	0(0)			
Yes	420	332(79.0)			
Timeliness of NIP vaccines			0.120		
Yes	440	321(73.0)			
No	9	9(100)	.		
Distance from home to immunization clinics (km)			0.244		
< 1	33	27(81.8)			
1–4.9	346	248(71.7)			
≥ 5	71	56(78.9)			
Time spent on each round-trip of vaccination (hours)			0.910		
< 0.5	107	77(72.0)			
0.5–0.9	203	150(73.9)			
≥ 1	140	104(74.3)			

*aOR* adjusted odds ratio

*NIP* National Immunization Program

^a^Calculated using Fisher’s exact test

Overall, 332(73.6%) of participating children were vaccinated with dose-1 Hib vaccine. Bivariate analyses showed that Hib vaccine uptake was positively associated with resident district (*P* = 0.021), mother being non-health workers (*P* = 0.010), father being non-health workers (*P* = 0.024) and parents being aware of Hib (*P* = 0.036). A higher likelihood of vaccination receipt was found among children whose parents being informed for Hib vaccination (Fisher’s exact test *P* < 0.001). There was a difference in Hib vaccination among districts. In multivariate analyses, mother being non-health workers (aOR = 2.33, 95% CI: 1.32–4.13, *P* = 0.004) and parents being aware of Hib (aOR = 1.75, 95% CI: 1.12–2.74, *P* = 0.013) remained significant (Table 2).

Sources of information regarding Hib for participants are listed in Table 3. For the 201 participants who were aware of Hib, the top five most frequently stated sources were vaccination personnel (76.6%), immunization promotional materials (39.3%), Internet (28.9%), journals and books (18.9%) and television programs (16.9%).

**Table 3:** Sources of information on Hib for participants (*n* = 201)

	Number of caregivers (%)
Communication with vaccination personnel	154(76.6)
Immunization promotion posters, brochures, etc.	79(39.3)
Internet	58(28.9)
Journals and books	38(18.9)
TV	34(16.9)
Broadcasting	28(13.9)
Friends or neighbors	18(9.0)
Others	2(1.0)

Note: participants were allowed to choose more than one

## Discussion

There were improved coverage and timeliness of dose-1 Hib vaccination and series completion in Shanghai City during the past decade. Surveillance in 2014 demonstrated that Shanghai ranked first in the average vaccinated doses (per 10,000 population) for Category II Vaccine in China [10]. The coverage of dose-1 Hib vaccination reached 65.5% among children born during 2012–2016, a substantial increase from 53.2% for the 2008–2010 birth cohort^13^. This was higher compared to the 2014 national estimate of 46.7% and that for children born in Shandong Province in 2009–2013 (60.8%) [10, 14], but was lower compared with those of Shenzhen (71.9%) and Maoming (76.1%) of Guangdong Province [15, 16].

There have been little data about the timeliness of dose-1 Hib vaccination in China. Timeliness of vaccine initiation is believed to be a better indicator of timely health care utilization. Timely vaccination is critical to protecting children in early life when they are at high risk of Hib invasive diseases. Even though we did observe an increase in timely administration of dose-1 Hib vaccine from 11.3% in the 2008–2010 cohort to 29.4% in the 2012–2016, more than two-thirds of children in Shanghai were not provided with timely immunological protection during their infancy.

Vaccine series completion is a key indicator of adequate immunological protection. In 2014, an average of 2.04 doses of Hib vaccine were given to each immunized child in China [10]. However, the number of doses is a poor marker for completion. In this study, we used the completion of Hib vaccination according to the age of receiving dose-1. Our study found that about half of children achieved series completion for Hib vaccination. By the end of 2016, 191 of the World Health Organization member states had implemented a routine immunization with Hib vaccine, and the global coverage with 3 doses of Hib vaccine is estimated at 70% [17]. Despite the recent improvement in initiation, completion, and timeliness reported in this study, these rates need to be further improved to provide adequate protection against Hib invasive disease among children.

Similar to the results from previous studies, [13, 14, 18] non-local children had worse vaccination outcomes than their local peers. The association between the completion of Hib vaccination and the residential district was significant in Jiading district but not in Jing’an and Changning, and the completion rate was higher in Jiading than in the other two districts. These disparities underscore the need for further interventions in vaccine promotion, targeting non-local children living in both developed and less-developed areas of Shanghai City.

Fewer than half (44.6%) of caregivers interviewed in our study were aware of *Haemophilus influenzae* type b, lower than that (55.2%) of a survey conducted in Hangzhou [19]. Caregivers’ awareness of Hib and being informed of the availability of Hib vaccine were understandably positively related to vaccine uptake in children [19]. The sources for caregivers to acquire Hib knowledge were relatively limited, mostly through communications with immunization clinics staff, reading promotional materials and the Internet. Thus, future educational programs should make use of these media to reach more caregivers. It is well acknowledged that health care professionals are the most trusted source of information on vaccination for parents [20, 21] However, we found that children of parents engaged in health industries were less likely to receive vaccination against Hib. This surprising finding indicates a lack of trust in Hib vaccines, particularly in healthcare personnel. A previous study among healthcare workers from Belgium has shown that the influenza vaccine coverage was negatively associated with misconceptions about influenza and its vaccine and underestimation of the disease risk [22]. Another study further found lower compliance in healthcare workers with new vaccination for their child, which had not been included in the immunization program [23]. Our finding calls for better education of healthcare personnel and emphasizes the importance of awareness about the risk of Hib.

This study has some limitations. First, our birth cohort was obtained from the SIPIS and might under-represent non-local children population living in Shanghai City, and as a result, the vaccination outcomes could be overestimated. Second, the cross-sectional design limited causal inferences for influencing factors associated with vaccine uptake. Third, the respondents were enrolled from the immunization clinics, and selection bias might occur. Finally, our findings from Shanghai City may be generalized to other metropolitan regions but not to rural areas and remote regions in China.

## Conclusions

This study provided an update on the coverage of dose-1 Hib vaccine, timeliness, and completion and explored factors associated vaccine uptake among children in Shanghai City. There were improvements in Hib vaccination, the measures varied with the status of residence and residential area. Hib vaccine uptake was associated with parental awareness of Hib and Hib vaccination as well as maternal occupation. The findings of this study highlighted sub-populations that should be targeted for future interventions. The inclusion of Hib vaccine into the national immunization program would substantially improve the coverage.
